# The prevalence of metabolic syndrome components, individually and in combination, in male patients admitted with acute coronary syndrome, without previous diagnosis of diabetes mellitus

**DOI:** 10.3402/ljm.v8i0.20185

**Published:** 2013-03-19

**Authors:** Rafid Fayadh Al-Aqeedi, Waleed Khalid Abdullatef, Wafer Dabdoob, Abdulbari Bener, Hajar A. Albinali, Abdurrazzak Gehani

**Affiliations:** 1 Department of Cardiology and Cardiovascular Surgery, Coronary Care Unit, Heart Hospital, Hamad Medical Corporation, Doha, Qatar; 2 Department of Medical Statistics and Epidemiology, Hamad Medical Corporation, Doha, Qatar; 3 Weill Cornell Medical College, Doha, Qatar

**Keywords:** male, metabolic syndrome, acute coronary syndrome, diabetes mellitus

## Abstract

**Background:**

Mortality from cardiovascular disease in the Middle East is projected to increase substantially in the coming decades. The prevalence of metabolic syndrome (MS) in acute coronary syndrome (ACS) continues to raise interest, but data from the Middle East is limited, especially in non-diabetic patients. This study was conducted to ascertain the prevalence of MS and frequency of its components, individually and in combination, in a male population presenting with ACS, but without a previous diagnosis of diabetes mellitus (DM).

**Methods:**

This is a prospective study of 467 consecutive male patients hospitalized for ACS. They were categorized according to the specific criteria stated in the latest joint statement for the global definition of MS.

**Results:**

The mean age was (49.7±10.7 years). Of the 467 patients, 324 (69.4%) fulfilled the criteria for MS. ST-Elevation Myocardial Infarction (STEMI) was identified in 178 patients (54.9%), and non-ST elevation ACS (NSTE-ACS) in 146 patients (45.1%). These proportions were not significantly different from those without MS (STEMI 51.7% vs. NSTE-ACS 48.3%, respectively). However, patients with MS were older (50.6±10 vs. 47.9±11 years; *p*=0.012), and more than half of those with MS were above 50 years. The most common abnormal metabolic components were reduced high-density lipoprotein cholesterol (HDL-c; 94.1%), elevated fasting blood glucose (FBG; 89.8%), and elevated triglycerides (81.8%), followed by increased waist circumference (61.7%) and raised blood pressure (40.4%). The majority of patients with MS had three or more metabolic components (326 patients, 69.4%), and 102 (21.8%) had two components, but only 37 (8.4%) had a single component.

**Conclusions:**

In ACS patients, without previous history of DM, MS is highly prevalent. Reduced HDL, elevated FBG and triglycerides were the most frequent metabolic components. The majority had multiple components. These findings raise alarm and show that drug therapy alone may not be fully effective, unless the underlying risk factors causing MS, such as weight and exercise, are also tackled.

Metabolic syndrome (MS) is a cluster of risk factors thought to contribute to the pathogenesis of atherosclerosis. The reported prevalence of MS among patients with acute coronary syndrome (ACS) varies between 29 and 62% (1–7). There is only limited data about the prevalence of MS in patients with ACS, particularly in the Middle-East populations. Furthermore, most studies have mixed diabetic and non-diabetic populations. In fact, they often have predominantly diabetic patients.

This prospective study was designed to focus on the prevalence of MS in patients admitted with ACS, but without a history of diabetes mellitus (DM), a population that has received less attention than those with DM. We also decided to concentrate on male subjects, as the majority of females admitted with ACS have DM. Furthermore, some of the components of MS are different for male and female subjects.

The criteria of MS were surrounded by differences among world authorities. In an attempt to eliminate some of the confusion, the latest joint statement was developed by a conglomerate of international organizations, including the International Diabetes Federation (IDF), the National Heart, Lung and Blood Institute (NHLBI), the World Heart Federation, the International Atherosclerosis Society, and the American Heart Association (AHA). These organizations have agreed on specific criteria for the global diagnosis of MS. This definition places major emphasis on central obesity in this condition. The criteria adopted for increased waist circumference are based on population and country specific definitions (8). Therefore, we sought to apply these criteria on a prospective hospital-based study of male patients in a Middle Eastern country who had ACS. The present study aims to ascertain the prevalence of MS and the frequency of its components individually and in combinations, in this specific male population.

## Methods

### Study design and population

In this prospective hospital-based study, we enrolled a total of 467 consecutive male patients who attended the emergency department and who were admitted to the Coronary Care Units, Hamad Medical Corporation, Doha, Qatar, with the diagnosis of ACS. Qatar has a population of around 1,720,000 according to the 2011 Qatar Statistics Authority (9).

The study was approved by the review board of the research committee in our institution prior to patient enrolment. Each participant was provided with full information about the study and was assured of strict confidentiality. Only participants who consented to participate were included in the study. Standard definitions were used to diagnose ACS in patients who had acute myocardial infarction (MI) (10) or unstable angina (11). Those who had acute MI were defined by a positive serial troponin-T blood test result (≥0.1 ng/mL) in the setting of symptoms and electrocardiographic changes consistent with either ST-elevation MI (STEMI) or non ST-elevation MI (NSTEMI). Unstable angina was diagnosed if the patient had a negative troponin blood test, but had any one of the following characteristics: new-onset angina (<2 months) of at least class III according to the Canadian Cardiovascular Society, prolonged (>20 min) angina at rest, recent (<2 months) worsening of angina pectoris, or angina that occurred within 2 weeks of an acute MI (11). All patients who were labeled with unstable angina but later found to have an alternative diagnosis were excluded. Each participant was prospectively interviewed during their first day of admission to ascertain lack of history of DM. Patients’ medical records and medications were also scrutinized for evidence of a previous diagnosis of DM. Only patients who lacked evidence of DM were deemed eligible to participate in the study.

Anthropometric measurements were obtained during admission for all patients. Height was measured in centimeters while the subject was standing in bare feet and with normal straight posture. Weight was measured in kilograms first thing in the morning before breakfast and body mass index (BMI) was calculated. Waist circumference was measured in centimeters atop the iliac crests before breakfast. Blood pressure was an average of at least two values automatically taken in the right upper arm during 1 h on first day of admission.

In the present study, we adopted the latest joint statement for the definition of MS, which identifies the global criteria based on population and country specific definitions for elevated waist circumference (Appendix 1, 2) (8, 12, 13).

A single set of cutoff points were used for all components except waist circumference. National or regional cutoff points for waist circumference were used. Male patients were diagnosed as having MS if they had any three of the following five components:

1. abdominal obesity (waist circumference >94 cm for Middle East and Mediterranean or >90 cm for Asians);
2. elevated triglyceride levels ≥1.7 mmol/L (150 mg/dL) or on drug treatment for hypertriglyceridemia;
3. reduced high-density lipoprotein-cholesterol (HDL-c) levels <1.0 mmol/L (40 mg/dL) or on drug treatment for low HDL-c;
4. repeated readings of elevated blood pressure (systolic ≥130 and/or diastolic ≥85 mm Hg) taken under standard conditions, or on active treatment for hypertension;
5. elevated fasting glucose levels ≥5.6 mmol/L (100 mg/dL).

Three sets of serial blood samples were obtained by vein puncture from all patients for cardiac biomarkers, including troponin-T and creatine kinase-MB. Blood was also drawn after a minimum of 12 h of fasting to measure triglycerides, HDL-c, and FBG. All biochemical parameters were assayed on Vitalab Selectra-E–Clinical Chemistry Analyzer.

Although the primary aim of this study was to describe the prevalence of various MS components among male patients who presented with ACS without a previous diagnosis of DM, secondary analyses were also performed for the socio-demographic and clinical characteristics, such as age, BMI, smoking, type of ACS, and the findings were compared between patients with and without MS.

### Statistical methods

Data were analyzed by using the Statistical Package for Social Sciences (SPSS, version 15.0). Both descriptive and analytic statistics were used in the data analysis. For categorical variables, basic descriptive statistics are reported as numbers and percentages.

Differences in the distribution of selected characteristics between groups with MS or without MS were examined by using chi-square tests of statistical significance for categorical variables. A two-tailed paired t-test was used to compare normal continuous variables. Fisher's exact test was used to compare discrete measures and proportions. A *p*-value of less than 0.05 was considered statistically significant. For the purpose of easier analysis, non-ST elevation MI (NSTEMI) and unstable angina (UA) were grouped together in a common group and labeled NSTE Acute Coronary Syndrome (NSTE-ACS), as opposed to ST-elevation MI (STEMI). This has been used by several recent studies (14, 15).

## Results

The study population consisted of 467 men admitted with ACS. The mean age (±SD) was 49.7±10.7 years. A total of 324 (69.37%) patients fulfilled the criteria stated in the latest joint statement for the definition of MS (8). Among them, STEMI was present in 178 (54.9%) patients and NSTE-ACS in 146 (45.1%) patients (*p*-value, non-significant).

Table 1 shows the distribution of patients according to their socio-demographics and clinical characteristics. ACS patients with MS were older than patients without MS (50.6±10 vs. 47.9±11; *p*=0.012). More than half of the patients with MS were older than 50 years, as compared to those without MS (53.7% vs. 46.9%, *p*<0.001). On the contrary, younger patients with MS (<40 years) made up a significantly less proportion than those without MS (12% vs. 27.3%, *p*<0.001).

**Table 1:** Socio-demographics and clinical characteristics of 467 male patients with (324 patients) or without (143 patients) metabolic syndrome

		ACS patients		
			
Variable	Total, *n* (%)	Patients with metabolic syndrome, *n* (%)	Patients without metabolic syndrome, *n* (%)	*p*
Number of patients	467 (100)	324 (69.3)	143 (30.6)	
Age in years (mean±SD)	49.7±10.7	50.6±10.2	47.9±11.8	0.012
Age group in years				
<40	78 (16.7)	39 (12.0)	39 (27.3)	<0.001
40–49	148 (31.7)	111 (34.3)	37 (25.9)
50–59	182 (39)	133 (41)	49 (34.3)
≥60	59 (12.6)	41 (12.7)	18 (12.6)
BMI, kg/m^2^ (mean±SD)	26.7±4.3	27.5±4.3	24.9±3.5	<0.001
Patients according to BMI				
Normal (<25 kg/m^2^)	175 (37.5)	90 (27.8)	85 (59.4)	<0.001
Overweight (25–30 kg/m^2^)	205 (43.9)	160 (49.4)	45 (31.5)
Obese (>30 kg/m^2^)	87 (18.6)	74 (22.8)	13 (9.1)
Current smokers	174 (37.3)	122 (37.7)	52 (36.4)	0.836
Blood pressure (mm Hg)				
Systolic (mean±SD)	117.4±18.6	119.1±19.7	113.3±14.8	0.002
Diastolic (mean±SD)	69.0±12.6	70.2±13.6	66.2±9.2	0.002
Waist circumference cm (mean±SD)	95.0±11.5	97.8±10.3	88.4±11.5	<0.001
Lipid profile mmol/L				
Total cholesterol	4.8±1.3	4.9±1.3	4.6±1.3	0.014
High density lipoprotein (mean±SD)	1.0±0.3	0.9±0.3	1.1±0.3	<0.001
Low density lipoprotein (mean±SD)	3.1±1.3	3.1±1.3	2.9±1.2	0.071
Triglycerides (mean±SD)	1.9±1.3	2.2±1.4	1.2±0.5	<0.001
Fasting blood sugar mmol/L (mean±SD)	7.0±2.6	7.2±2.5	6.3±2.7	<0.001
Acute coronary syndrome				
STEMI	252 (54)	178 (54.9)	74 (51.7)	0.294
NSTE-ACS	215 (46V)	146 (45.1)	69 (48.3)
Metabolic syndrome components				
Elevated waist circumference	303 (64.9)	265 (81.8)	38 (26.6)	<0.001
Elevated triglycerides	216 (46.3)	200 (61.7)	16 (11.2)	<0.001
Reduced HDL cholesterol	407 (87.2)	305 (94.1)	102 (71.3)	<0.001
Elevated blood pressure	150 (32.1)	131 (40.4)	19 (13.3)	<0.001
Elevated fasting glucose	359 (76.9)	291 (89.8)	68 (47.6)	<0.001

Data are expressed as number (%) of patients unless otherwise indicated.

ACS: acute coronary syndrome, BMI: body mass index, STEMI: ST-elevation myocardial infarction, NSTE-ACS: non-ST elevation-acute coronary syndrome, HDL-c: high-density lipoprotein cholesterol.

Patients with MS had a higher prevalence of increased BMI as compared to the group without MS (BMI means 27.5±4.3 vs. 24.9±3.5; *p*<0.001). As for obesity (>30 kg/m^2^), the prevalence was 22.8% versus 9.1%, respectively (*p*<0.001). The overall prevalence of smoking in our cohort was 37.3% and there was no significant difference in the prevalence of current smokers between those with and without MS: 122 (37.7%) versus 52 (36.4%), respectively (*p*=0.836). There was also no significant difference in the prevalence of different types of ACS between the MS and non-MS groups (STEMI, 54.9% vs. 51.7%; NSTE-ACS, 45.1% vs. 48.3%, respectively; *p*=0.294 for both).

**Fig. 1:**
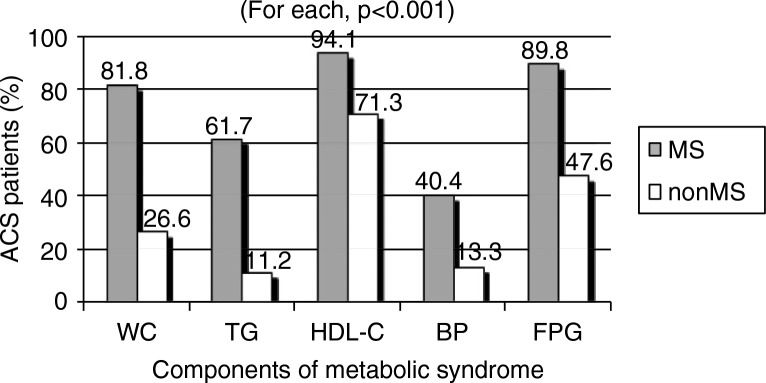
Prevalence of metabolic components in male patients presenting with acute coronary syndrome with or without metabolic syndrome. Data are expressed in percentage of patients. ACS: acute coronary syndrome, MS: metabolic syndrome, non-MS: non-metabolic syndrome, WC: elevated waist circumference, TG: elevated triglyceride, HDL-c: reduced high-density lipoprotein-cholesterol, BP: elevated blood pressure, FPG: elevated fasting plasma glucose levels.

The prevalence of each of the metabolic components among patients with or without MS is shown in Fig. 1. The reduced HDL-c observed in 305 patients (94.1%) and the elevated fasting blood glucose (FBG) in 291 patients (89.8%) were the most common metabolic components. The next most frequent components were increased waist circumference in 265 patients (81.8%), elevated triglyceride in 200 patients (61.7%) and raised blood pressure in 131 patients (40.4%). The mean value of HDL-c was significantly lower in MS than in non-MS patients (0.9±0.3 vs. 1.1±0.3; *p*<0.001). While triglycerides mean value was significantly higher in MS than in non-MS patients (2.2±1.4 vs. 1.2±0.5; *p*<0.001), the mean value for FBG was significantly higher in the MS than in the non-MS group (7.2±2.5 vs. 6.3±2.7; *p*<0.001). The mean systolic and diastolic blood pressure values were slightly but significantly higher in patients with MS than in those without MS (systolic, 119.1±19.7 vs. 113.3±14.8, respectively; diastolic, 70.2±13.6 vs. 66.2±9.2, respectively; *p*=0.002 for both).

**Fig. 2:**
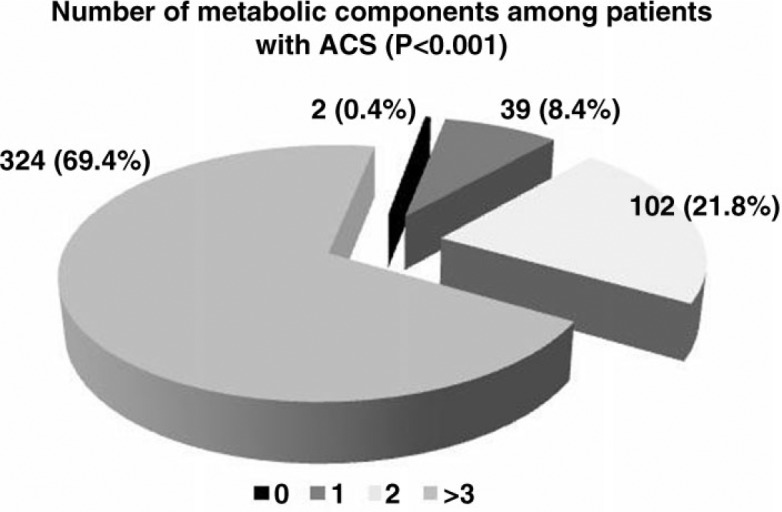
Individual metabolic abnormalities among a male population with acute coronary syndrome (*N*=467). Data are expressed as number (%) of patients. ACS: acute coronary syndrome.

In patients with ACS, the majority (324 patients, 69.4%) had three or more (3–5) components. Less than one-third (143 patients, 30.6%) had two or fewer components of MS (Fig. 2). Only 39 patients (8.4%) had a single component of MS.

## Discussion

MS consists of a cluster of metabolic disorders, many of which promote the development of atherosclerosis and increase the risk of cardiovascular diseases. Western studies suggest that MS is very commonly associated with coronary artery disease (2). However, there is very limited information about the prevalence of MS in ACS patients in the developing world, particularly in the Middle East. To the best of our knowledge, this is the first prospective study from the Middle East region to address the prevalence of MS in patients presenting with ACS without a previous diagnosis of DM.

Because of the previous diversity in the definition of MS criteria, the reported prevalence of MS in patients with ACS ranges between 29 and 34% when known diabetics where excluded (1, 4), and between 38 and 62% in studies where diabetic patients were included (2, 3, 5–7). Analyzing some of the studies that evaluated the prevalence of MS in ACS before the latest joint definition of MS reveals interesting discrepancies. In a study using the criteria of the National Cholesterol Education Program-Adult Treatment Panel III (NCEP ATP-III), the prevalence of MS was 46% in a cohort of 633 patients with STEMI (2). Milani et al. assessed 235 consecutive patients after a coronary event and reported a prevalence of 62% (6), while a Canadian study reported a prevalence of MS in up to 51% (16).

In the present study, we show that the prevalence of MS in male patients hospitalized with ACS was 69.37%, when MS is defined according to the latest joint statement criteria. This is considerably higher than some other studies, especially bearing in mind that our patients did not have a history of diabetes. This could be attributed to several factors. First, the reported prevalence rate of MS in the general population of 35.4% (using the IDF definition of MS), which is considerably higher in Qatar than that reported in nearby Gulf and Western countries (17). Second, the cutoff of waist circumference of the latest global definition that we applied is lower than that used by previous reports adopting the criteria of the NCEP/ATP III in 2001 (12), or the criteria of the AHA/NHLBI in 2005 (18), which applied a waist circumference of ≥102 cm in men. These factors may, in part, explain the higher prevalence of MS in the present study.

There was no significant difference in the prevalence of different types of ACS between groups with and without MS. Among the 467 patients studied, 54% had STEMI and 46% had NSTE-ACS. With regard to age, it is worth noting that MS prevalence peaked at 53.7% in persons older than 50. A similarly high prevalence of the MS in AMI patients (46%) among older age groups was also reported by Zeller et al. (2). In contrast to the older age group (>50 years), the prevalence of MS among the younger age group (<40 years) showed a significant lower proportion with than without MS (12% vs. 27.3%, respectively; *p*<0.001). This may point to other overriding covariates like dyslipidemia or sub-clinical inflammation, which might play a stronger role in younger patients. This mandates further investigation. Obesity and being overweight were significantly more prevalent in the MS group, a finding similar to results reported from neighboring countries on populations even without ACS (19, 20).

The majority of our patients had three or more components (69.4%). Less than one-third (30.6%) had two or fewer components of MS. This indicates that not only is MS prevalent, but if severity could be implied by the number of components, most patients had a severe form also. Among the individual components of MS, we found reduced HDL-c to be the most common component (87.2%), followed by elevated FBG (76.9%). Most patients had not had an encounter with physicians before, and for many the admission with ACS was the first such encounter. Therefore, almost all patients did not have a glucose tolerance test or HbA1c measurement before this admission. This underscores the importance of public screening programs, especially in communities with high prevalence of DM.

The overall findings of the study underscore the need for lifestyle modification, particularly in view of the living environment in some Middle Eastern countries, which is witnessing fast economic development and urbanization, in addition to a hot climate which might discourage outdoor activities and instead encourage a sedentary life. It is worth emphasizing that while some components of MS can be treated by medication, there are components that are much easier and more effectively controlled by improved diet and exercise. Hence, national and regional health and nutrition education programs of the population are needed, and the role of health institutions in this regard is critical.

## Conclusion

MS has become a global and major public health problem. The present study reveals a high prevalence of MS (67.92%) in male patients with ACS but without a previous diagnosis of DM. The majority had three or more components of MS. Reduced HDL-c was the most common metabolic abnormality While there is often emphasis on drug therapy to treat MS, our findings suggest that this may not be adequate, unless other important measures, like diet and physical activity, are also emphasized in population campaigns.

## Appendix

**Appendix 1:** Criteria for clinical diagnosis of the metabolic syndrome (8) applied to the 467 male patients studied.

Measure	Categorical cutoff points
Elevated waist circumference*	Population- and country-specific definitions
Elevated triglycerides (drug treatment for elevated triglycerides is an alternate indicator†)	≥150 mg/dL (1.7 mmol/L)
Reduced HDL-c (drug treatment for reduced HDL-c is an alternate indicator†)	<40 mg/dL (1.0 mmol/L) in males; <50 mg/dL (1.3 mmol/L) in females
Elevated blood pressure (antihypertensive drug treatment in a patient with a history of hypertension is an alternate indicator)	Systolic ≥130 and/or diastolic ≥85 mm Hg
Elevated fasting glucose‡ (drug treatment of elevated glucose is an alternate indicator)	≥100 mg/dL

HDL-c: high-density lipoprotein cholesterol.

* It is recommended that the IDF cut points be used for non-Europeans and either the IDF or AHA/NHLBI cut points used for people of European origin until more data are available.

† The most commonly used drugs for elevated triglycerides and reduced HDL-c are fibrates and nicotinic acid. A patient taking one of these drugs can be presumed to have high triglycerides and low HDL-c. High-dose omega-3-fatty acids presume high triglycerides.

‡ Most patients with type 2 diabetes mellitus will have metabolic syndrome according to the proposed criteria.

**Appendix 2:** Current recommended waist circumference thresholds for abdominal obesity by organization (8).

	Recommended waist circumference threshold applied for abdominal obesity		
	
Population	Organization (Reference)	Men (cm)	Women (cm)
Asian (including Japanese)	IDF (12)	≥90	≥80
Asian	WHO (13)	≥90	≥80
Middle East, Mediterranean	IDF (12)	≥94	≥80

IDF: International Diabetes Federation, WHO: World Health Organization.
